# Use of the Children’s Observation and Severity Tool (COAST), an Adaptation of the Paediatric Early Warning Score, in the Emergency Department as a Predictor for Hospital Admission: A Retrospective Cohort Study

**DOI:** 10.3390/children12020136

**Published:** 2025-01-26

**Authors:** James Truell, Sara Neville-Smith, Hayley Hutton

**Affiliations:** Chelsea & Westminster NHS Foundation Trust, London SW10 9NH, UK; s.neville-smith@nhs.net (S.N.-S.); hayley.hutton1@nhs.net (H.H.)

**Keywords:** paediatric, emergency, observation tool, admissions

## Abstract

Background: Paediatric Early Warning Scores (PEWS) are designed to detect the clinically unwell or deteriorating child within a hospital setting. There is a drive within the NHS to standardise this scoring system across various settings. According to the Royal College of Paediatrics and Child Health, there is currently a national review on triage tools used within emergency departments; however, evidence to date is scarce. Within the Chelsea and Westminster NHS Foundation Trust, we utilise both the Manchester Triage System (MTS) and the Children’s Observation and Severity tool (COAST), an adaption of PEWS, in our Paediatric Emergency Department (PED). Methods: This retrospective cohort study is the largest of its kind and was performed to validate COAST, compare it to MTS and determine if it is a good predictor of hospital admission. Demographic data, initial MTS scores, initial COAST scores and admission outcomes of 41,030 paediatric emergency department attendances were analysed, encompassing 27,196 unique patients. Results: Results demonstrated that high COAST scores on arrival are strongly correlated with hospital admission, with positive predictive values of 59.52% with COAST of ≥3 and 100% for with score threshold of ≥5. In comparison with the MTS, COAST is better at predicting admission. Conclusions: We conclude that COAST performs well in correlating to and thus predicting paediatric hospital admission outcome from the PED.

## 1. Introduction

In 2018, NHS England, in conjunction with the Royal College of Paediatric and Child Health and the Royal College of Nursing, began a project to develop a national early warning scoring system for paediatric observations [1]. The aim was to develop a standardised system-wide program that allows for prompt recognition of the deteriorating child within healthcare settings. Appropriate escalation strategies are then triggered, which improve communication between teams and ultimately have a positive impact on improving patient outcomes and reducing adverse events. The hypothesis is that a standardised system would help to alleviate inconsistencies between English hospital sites. This should reduce challenges for staff moving between hospitals by ensuring all wards are using the same observation charts [2]. In November 2023, NHS England published the outcome of this collaboration as the National Paediatric Early Warning System (nPEWS) observation and escalation charts [3].

The nPEWS charts are intended for use on inpatient wards. In January 2024, the Royal College of Emergency Medicine (RCEM) released a statement warning that the nPEWS chart is not validated for use in the emergency department. It states that there may be other scoring systems which are better at quickly and reliably identifying those children who are likely to require admission [4,5,6]. This provided the stimulus for this project.

Previous studies and literature reviews have evaluated a range of escalation and observation tools within various hospital settings [7,8,9,10]. These show the wide heterogenicity in the use of paediatric triage and escalation tools used both in England and internationally, but they can be useful in escalating care for unwell children. As such, RCEM recommends that each Paediatric Emergency Department (PED) use an early warning score they consider most appropriate for their service [6].

The Chelsea and Westminster Hospital NHS Foundation Trust is a busy hospital based in Northwest London, serving a population of more than 1.5 million people [11]. It has a separate specialist PED and is a training site for sub-specialty Paediatric Emergency Medicine trainees.

Since 2014, Chelsea and Westminster Hospital PED has been utilising two tools to highlight patients requiring urgent treatment and identify children presenting with severe illness. Originally developed by the NHS Institute for Innovation and Improvement, a set of adapted PEWS charts called the Children’s Observation and Severity Tool (COAST) is used [5]. The COAST scoring system strives to rapidly determine which children may require enhanced medical attention based on an initial set of clinical observations. The Manchester Triage system is a validated tool which is also used at Chelsea and Westminster for nurse-led triage and provides a useful comparator to COAST in this study [8,12].

A brief literature review by the authors unveiled only one paper evaluating COAST’s ability to predict hospital admission [5]. We feel that this small-cohort study did not provide enough evidence for our department to continue using COAST without further validation. It is important to note that the low evidence base for COAST is not unusual in the context of paediatric observation tools [10].

It is in the context of NHS England’s desired rollout of national PEWS charts and RCEM’s recommendation against them that the aim of this study is to evaluate the efficacy of COAST in predicting hospital admission for children on presentation to the emergency department. With this study, we hope to provide an improved evidence base for COAST that supports its continued use as a paediatric observation tool in the emergency department and to demonstrate that it is an effective tool to predict admission.

## 2. Materials and Methods

The site of this study was Chelsea and Westminster Hospital in London, England. Data was collected from 1 September 2023 to 1 September 2024. Data were pulled from Cerner, our electronic patient record system in use, in which nursing staff record a triage (MTS) score on arrival in PED and a COAST score based on a patient’s initial set of observations. Data were analysed using Microsoft Excel and statistical software [13]. For this study, only the first COAST score entered was chosen, as subsequent scores may have been altered by medical management or other changes in circumstances. For patients with multiple attendances, we analysed the first COAST score of each discrete attendance.

The end metric chosen was admission to either Chelsea and Westminster Hospital or transferred to another inpatient unit. All other discharge outcomes were classed as ‘not admitted’. Of important note, in Chelsea and Westminster PED, it is at the nurses’ discretion to take a set of observations for the following presentations: Assault (minor), Bites, Burns (minor), Dislocations, Ear, Nose and Throat Injuries, Eye Injuries, Fractures, Head Injuries (minor), Ingestions, Limb Problems, Limps, Plaster/Dressing problems, Soft Tissue Injuries and Wounds. This accounts for the large number of ‘not recorded’ results below.

Table 1 shows the COAST scoring criteria used by the nursing staff, giving a minimum score of 0 and a maximum score of 6. Table 2 shows the normal limits used for scoring heart rate and respiratory rate. For behaviour, the AVPU scale is used, assessing whether the child is Alert (A), responds to Voice (V), responds to Pain (P) or is Unresponsive (U).

Admission outcomes for initial COAST ≥ 1 to ≥5 were used to calculate sensitivity, specificity, positive predictive values (PPV), negative predictive values (NPV), positive likelihood ratios and negative likelihood ratios with 95% confidence intervals using Excel and statistical software. This is consistent with similar research on PEWS [7]. False positive rates were also calculated (patients not admitted who had scores over the score threshold/all patients not admitted). Performance of increasing COAST score thresholds are discussed below.

The Manchester Triage System (MTS) is used in triage, as explained in Table 3. It uses standardised signs and symptoms to assess patients attending the emergency department, aiming to ensure patients are assessed in order of clinical priority [12]. We have analysed data from the same patient cohort to assess performance of MTS scores in predicting patient admissions and to compare to values obtained for COAST analysis.

## 3. Results

In total, 41,031 PED attendances were reviewed, attributed to 27,197 unique patients. A single anomalous result, a COAST score of 8, was removed from analysis due to administration error (the highest possible COAST score is 6). Therefore 41,030 attendances of 27,196 unique patients were analysed. 

2960 patients were admitted, giving an overall admission rate of 7.2%. 38,070 patients were not admitted. Those not admitted include 1658 patients who left before treatment was complete. Also included in the not admitted category are the 504 patients who were streamed to other services: 499 were sent to general practitioners, dentists or an urgent care centre, and 5 were sent to other facilities. We do not know their admission status after leaving our department. Additionally, 4 patients were dead on arrival to PED and did not have COAST scores recorded.

Table 4 shows the demographic breakdown of the patient population. A significant proportion of the patients were below the age of 5 (58.9%). 55.2% of patients were male. The varied ethnic diversity of the cohort reflects the central London population, allowing this study to be more widely generalised.

The relationship between initial COAST scores and admission rates is detailed in Table 5, where a clear trend emerges. There is a strong positive correlation between admission rate and increasing initial COAST: from 6.6% of patients who were admitted scoring 0, to 100% of patients who were admitted scoring 5.

Table 6 groups together the data for COAST scores (≥1 to ≥5) to allow for a more detailed examination of admission outcome against increasing COAST score thresholds. For example, ≥3 means all children scoring 3, 4, 5 or 6.

To evaluate the predictive accuracy of COAST regarding admission, key analytic metrics of performance were calculated for each threshold, as presented in Table 7 and Table 8. At a threshold of ≥1, sensitivity was 51.81% and specificity 69.33%, with a positive predictive value of 15.77% and high negative predictive value of 93.36%. Sensitivity decreases with increasing thresholds of COAST, falling to less than 10% at scores ≥3, while specificity sharply rises with increased COAST scores. At COAST ≥ 2, the specificity for capturing admissions is 93.83%, rising to greater than 99% at COAST of ≥3 and to 100% for a threshold of ≥5.

Likelihood ratios and predictive values further underscore this dynamic. Positive predictive values increased markedly with higher thresholds, increasing from 59.52% at COAST ≥ 3 to 81.44% at scores ≥4 and 100% at scores ≥5. In line with this, the likelihood of admission was shown to increase significantly with increasing initial COAST scores. An initial COAST ≥1 has a positive likelihood ratio of 1.75, increasing to 13.78 at initial scores ≥3, to 41.43 at initial scores ≥4 and peaking at a positive likelihood ratio over 140 with initial COAST ≥ 5. This strongly suggests that high initial COAST scores serve as a good indicator of hospital admission.

False positive rates decreased sharply with increasing COAST scores, from 30.67% with initial COAST ≥ 1 to 6.17% with initial COAST ≥ 2 to a less than 1% false positive rate with scores ≥3 and 0% with scores ≥5.

An analysis of MTS triage scores and their predictive accuracy for admission was conducted for comparison with the findings for COAST. Table 9 shows admission rates for increasing severities of triage scores: 5 (Non-urgent), 4 (Standard), 3 (Urgent), 2 (Emergency) and 1 (Immediate).

This showed a weak positive correlation between increasing MTS score and admission, where only 17.3% of patients scoring 2 and 17.8% of patients scoring 1 were ultimately admitted.

In performing analysis of MTS data (Table 10 and Table 11), scores were grouped in the same way as COAST data. As explained in Table 3, an MTS score of 5 is the least severe, whereas when using COAST scoring, a score of 0 is the least severe. Note that for ease of comparison, MTS data have been ordered in increasing severity (5 to 1). It was then grouped such that a score of ≥4 indicates a score of 4 or greater severity (‘standard’ priority and above, i.e., scores of 4, 3, 2 and 1), and a score of ≥1 indicates the highest severity only (immediate: life-threatening).

An MTS score of ≥4 has a sensitivity of 99.55%. This shows that virtually all patients scoring a 5 would not be admitted, with a low specificity of 2.68%. Sensitivity decreased while specificity increased with decreasing MTS score. Positive predictive values for admission increase with decreasing MTS scores. For MTS ≥4, the PPV is 7.36% (half the value obtained from COAST ≥ 1), tapering to at best a PPV of 17.83% for MTS scores ≥ 1. In comparison, a PPV of 100% for COAST ≥ 5.

Similarly, positive likelihood ratios for admission increased but less convincingly, at 2.79 for MTS of highest severity (compared to over 140 for highest severity using the COAST system, PEWS ≥ 5). False positive rates were significantly higher using MTS scores, at 97.32% with MTS ≥ 4, to 33.27% with MTS ≥ 3, to 22.78% with scores ≥ 2 and 1.25% with scores ≥ 1.

## 4. Discussion

COAST may not be a widely validated PED observation tool; however, this paper demonstrates that it performs remarkably well as a predictor of admission to hospital. Data analysed have shown a strong correlation between rising COAST scores and the probability of admission.

One or more abnormal observations as part of this scoring system (COAST ≥ 1) is a balanced indicator for capturing potential admissions while maintaining acceptable specificity. The sensitivity of lower initial COAST for reflecting the probability of admission should be interpreted within the broader patient picture, considering that patients are admitted for many reasons other than abnormal physiological observations; a diverse range of children are admitted to hospital, including those who may be admitted for psychiatric, safeguarding or non-acute surgical needs. Decreasing sensitivity of higher initial COAST scores reflects the fact that the majority of paediatric admissions are not in an extreme critical condition.

The analysis shows that while COAST cannot be used in isolation in patients with lower scores, its high specificity, positive predictive values and positive likelihood ratios with higher score thresholds (particularly with COAST ≥ 3) make it a useful tool in the arsenal of a PED. COAST effectively highlights those patients who require more urgent medical attention, triggering clinical reviews, prompting timely escalation to increasing clinician seniority, supporting clinical decision making and aiding in allocation of resources. Furthermore, patient flow and forward planning can be improved using COAST. Action can be taken earlier in children with high scores, such as booking ward beds or taking viral swabs required prior to admission.

Evaluating COAST against MTS shows that COAST is a far superior tool when predicting admissions from PED. MTS has higher false positive rates, lower positive predictive values and lower positive likelihood ratios. These findings show that higher-acuity MTS scores in triage do not correlate strongly to admission outcome. This is likely due to the role that the MTS plays within PED, which is to have swift clinician reviews of unwell patients. The high sensitivity of MTS is valuable in not missing any unwell children at the point of triage. As such, we are not advocating for replacing MTS with COAST. Both tools have a unique role within the PED environment, with MTS highlighting patients who need urgent review and COAST highlighting those likely to need admission.

We note some limitations to this paper. It is a single-centre, retrospective study. Nurse/parent concern is a subjective measurement which, although used in other tools, is open to user error [4]. Of 41,030 analysed attendances, 10,807 did not have COAST scores recorded, a significant loss of data for the study. We also acknowledge that there would be benefits from comparing COAST to other paediatric triage systems but we were constrained by the data available to us. We also recognise that no triage scoring system is perfect and triage does not replace the role of physician-led clinical assessment in an emergency department.

The large number of patients who did not have COAST scores recorded reflects the number of minor injuries that attend Chelsea and Westminster PED. With an admission rate of 0.4%, we feel that the departmental policy of not taking observations on these patients is justified. This policy saved valuable nursing time that can be utilized elsewhere, while not putting patients at significant risk of harm, but may have introduced a level of selection bias into our dataset.

This study is by far the largest of its kind, analysing 41,030 attendances of 27,196 patients, compared to the 1921 patients in the previous study of COAST and 18,073 patients of the largest similar study [5,9,10]. With the planned nationwide implementation of nPEWS this paper provides a vital baseline to compare the performance of nPEWS in PED. RCEM guidance is that it should be left up to individual PEDs to decide which observation tool they wish to use based on local practices and resources available [6]. As authors we strongly suggest that other PEDs consider the use of COAST within their departments as an effective observation tool and predictor of hospital admission.

## 5. Conclusions

In conclusion, this paper provides vital evidence in a poorly researched field, supporting the use of COAST in PED and proving its utility in predicting admissions. Continued and wider research is also required around the use of PED observation tools. It would also be useful to capture end patient outcomes in future research, such as morbidity, mortality and critical care admission.

## Figures and Tables

**Table 1:** COAST Scoring Criteria.

COAST Scoring Section	0 Points	1 Point
Nurse/Parent Concern	None	Yes
Respiratory Rate	Within Normal Limits	Outside Normal Limits
Respiratory Distress	Nil/Mild	Moderate/Severe
Requiring O2 ^1^	No	Yes
Heart Rate	Within Normal Limits	Outside Normal Limits
Behaviour (AVPU) ^2^	A	V, P, U

^1^ In most children with no respiratory co-morbidity, oxygen therapy is required in the event of hypoxia to maintain oxygen saturations > 92%. Other indications include shock, severe trauma, sepsis and anaphylaxis [14]. ^2^ Behaviour is classified according to the AVPU scale: whereby a patient’s neurological status/consciousness is assigned as Alert (A), responsive to Voice (V), Pain (P), or Unresponsive (U).

**Table 2:** COAST Heart Rate and Respiratory Rate Normal Limits, per age group.

Age	Heart Rate (Beats per Minute)	Respiratory Rate (Breaths per Minute)
<1	90–160	30–60
1–4	90–140	20–40
5–12	70–120	20–30
13–19	60–100	10–20

**Table 3:** Manchester Triage System (MTS).

MTS Score	Indicative Clinical Signs/Symptoms
1 (Immediate)	Airway compromise, inadequate breathing, stridor, drooling, shock, unresponsive.
2 (Very urgent)	Very low PEFR ^1^, very low SaO2, increased work in breathing, unable to talk in sentences, significant respiratory history, acute onset after injury, responds to voice or pain only, exhaustion.
3 (Urgent)	Low PEFR ^1^, low SaO2, inappropriate history, pleuritic pain.
4 (Standard)	Wheeze, chest infection, chest injury, recent problem.
5 (Non-urgent)	No features mentioned above.

^1^ Peak Expiratory Flow Rate (PEFR): the volume of air expelled from the lungs in one rapid and forceful exhalation.

**Table 4:** Demographics of attendances analysed.

Demographics	Sub-Section	Number	Percentage
Age	0	7599	18.5%
	1–5	16,557	40.4%
	6–10	9089	22.2%
	11–15	7786	19.0%
Sex	Male	22,664	55.2%
	Female	18,361	44.7%
Ethnicity	Other White	11,909	29.0%
	White British	8157	19.9%
	Asian	6767	16.5%
	Other	5595	13.6%
	Black	4272	10.4%
	Mixed	4048	9.9%
	Not stated	1618	3.9%

**Table 5:** COAST scoring system and admission rates.

Initial COAST Score	Admitted	Not Admitted	Admission Rate (%)
0	1346	18,932	6.6%
1	809	6693	10.8%
2	484	1497	24.4%
3	196	169	53.7%
4	64	18	78.0%
5	15	0	100%
Not Recorded	46	10,761	0.4%
Total	2960	38,070	7.2%

**Table 6:** Analysis—grouped initial COAST scores and admission numbers.

	Admitted		Not Admitted	
Initial COAST	COAST Score > Threshold	COAST Score < Threshold	COAST Score > Threshold	COAST Score < Threshold
≥1	1568	1346	8377	18,932
≥2	759	2155	1684	25,625
≥3	275	2639	187	27,122
≥4	79	2835	18	27,291
≥5	15	2899	0	27,309

**Table 7:** Analysis—performance characteristics of initial COAST scores in reflecting admissions.

Initial COAST	Sensitivity (95% CI)	Specificity (95% CI)	Positive Predictive Value (95% CI)	Negative Predictive Value (95% CI)
≥1	51.81% (51.98 to 55.63%)	69.33% (68.77 to 69.87%)	15.77% (15.27 to 16.28%)	93.36% (93.11 to 93.61%)
≥2	26.05% (24.46 to 27.68%)	93.83% (93.54 to 94.12%)	31.07% (29.45 to 32.73%)	92.24% (92.09 to 92.40%)
≥3	9.44% (8.40 to 10.56%)	99.32% (99.21 to 99.41%)	59.52% (55.08 to 63.82%)	91.13% (91.04 to 91.23%)
≥4	2.71% (2.15 to 3.37%)	99.93% (99.90 to 99.96%)	81.44% (72.48 to 87.97%)	90.59% (90.54 to 90.64%)
≥5	0.51% (0.29 to 0.85%)	100.00% (99.99 to 100%)	100.00% (78.20 to 100%)	90.40% (90.38 to 90.43%)

**Table 8:** Analysis (continued)—performance characteristics of initial COAST Scores in reflecting admissions.

Initial COAST	Positive Likelihood Ratio (95% CI)	Negative Likelihood Ratio (95% CI)	False Positive Rate
≥1	1.75 (1.69 to 1.82)	0.67 (0.64 to 0.69)	30.67%
≥2	4.22 (3.91 to 4.56)	0.79 (0.77 to 0.81)	6.17%
≥3	13.78 (11.49 to 16.53)	0.91 (0.90 to 0.92)	0.68%
≥4	41.13 (24.69 to 68.53)	0.97 (0.97 to 9.98)	0.07%
≥5	140.58 (18.58 to 1063.90)	0.99 (0.99 to 1.00)	0.00%

**Table 9:** Manchester Triage System (MTS) triage scoring and admission rates.

MTS Triage Score	Admitted	Not Admitted	Admission Rate (%)
5	13	1006	1.3%
4	611	24,003	2.5%
3	498	3930	11.2%
2	1688	8069	17.3%
1	102	470	17.8%
Not recorded	48	593	8.1%
Total	2960	38,071	7.2%

**Table 10:** Analysis—performance characteristics of triage MTS scores in reflecting admissions.

MTS Score	Sensitivity (95% CI)	Specificity (95% CI)	Positive Predictive Value (95% CI)	Negative Predictive Value (95% CI)
≥4	99.55% (99.24 to 99.76%)	2.68% (2.52 to 2.85%)	7.36% (7.34 to 7.38%)	98.72% (97.82 to 99.26%)
≥3	78.57% (77.04 to 80.05%)	66.73% (66.25 to 67.21%)	15.51% (15.20 to 15.82%)	97.57% (97.39 to 97.73%)
≥2	61.47% (59.67 to 63.24%)	77.22% (76.79 to 77.64%)	17.33% (16.84 to 17.83%)	96.27% (96.10 to 96.43%)
≥1	3.50% (2.86 to 4.24%)	98.75% (98.63 to 98.86%)	17.83% (14.95 to 21.13%)	92.94% (92.90 to 92.99%)

**Table 11:** Analysis (continued)—performance characteristics of triage MTS scores in reflecting admissions.

MTS Score	Positive Likelihood Ratio (95% CI)	Negative Likelihood Ratio (95% CI)	False Positive Rate
≥4	1.02 (1.02 to 1.03)	0.17 (0.10 to 0.29)	97.32%
≥3	2.36 (2.31 to 2.42)	0.32 (0.30 to 0.34)	33.27%
≥2	2.70 (2.61 to 2.79)	0.50 (0.48 to 0.52)	22.78%
≥1	2.79 (2.26 to 3.45)	0.98 (0.97 to 0.98)	1.25%

## Data Availability

The data presented in this study may be available on request from the corresponding author subject to patient confidentiality and applicable data protection and privacy laws.
